# Telecommunication and neuropsychiatric symptoms in long term care dementia patients during the COVID-19 lockdown ERA

**DOI:** 10.1192/j.eurpsy.2021.745

**Published:** 2021-08-13

**Authors:** A. Konstantinou

**Affiliations:** Old Age Psychiatry, Ippokrateio Therapeutirio, Larissa, Greece

**Keywords:** Telepsychogeriatrics, Long Term Care, dementia, Neuropsychiatric symptoms

## Abstract

**Introduction:**

Ippokrateio Therapeutirio in the city of Larissa, Thessaly, Greece is a private for-profit psychogeriatric hospital focusing on Long Term Care on patients with dementia. During the COVID-19 era lockdown visits by carers/relatives/friends were forbidden due to the preventive government measures. At that same time appearance and/or exaggeration of neuropsychiatric symptoms was observed. In order to restore communication issues we performed telecommunication sessions (videocalls) and measured, among other factors, neuropsychiatric symptoms before and after sessions.

**Objectives:**

Primary objective was to check for relations between video-calls and changes in neuropsychiatric symptoms using NeuroPsychiatric Inventory (NPI). Secondary objective was to check for carers and patients satisfaction, mainly through qualitative information.

**Methods:**

120 patients with diagnosis of minor or major neuroconitive disorder of any type participated in the video call sessions. Two video calls per patient took place (1 per week) with a 10-inches tablet. Neuropsychiatric Inventory (NPI) was performed before the start of the video-calls. NPI had been performed again the week after both sessions were completed. Satisfaction of carers and patients was recorded, mostly as qualitative data.

**Results:**

Neuropsychiatric symptoms improved in patients with mild or moderate neurocognitive decline. In more severe cases though anxiety, irritability and sleep problems worsened. Satisfaction reached almost 95% of the carers.

**Conclusions:**

Video calls could be a very good way to surpass the communication burden during the pandemic restrictions for LTC dementia patients. Caution should be given to severely demented patients since clinical observations show that a cluster of symptoms worsens.

